# Optimizing the thermoelectric performance of zigzag and chiral carbon nanotubes

**DOI:** 10.1186/1556-276X-7-116

**Published:** 2012-02-11

**Authors:** Xiaojian Tan, Huijun Liu, Yanwei Wen, Hongyan Lv, Lu Pan, Jing Shi, Xinfeng Tang

**Affiliations:** 1 Key Laboratory of Artificial Micro- and Nano-structures of Ministry of Education and School of Physics and Technology, Wuhan University, Wuhan, 430072, China; 2 State Key Laboratory of Advanced Technology for Materials Synthesis and Processing, Wuhan University of Technology, Wuhan, 430072, China

## Abstract

Using nonequilibrium molecular dynamics simulations and nonequilibrium Green's function method, we investigate the thermoelectric properties of a series of zigzag and chiral carbon nanotubes which exhibit interesting diameter and chirality dependence. Our calculated results indicate that these carbon nanotubes could have higher *ZT *values at appropriate carrier concentration and operating temperature. Moreover, their thermoelectric performance can be significantly enhanced via isotope substitution, isoelectronic impurities, and hydrogen adsorption. It is thus reasonable to expect that carbon nanotubes may be promising candidates for high-performance thermoelectric materials.

## Introduction

As it can directly convert waste heat into electric power, thermoelectric material is expected to be one of the promising candidates to meet the challenge of energy crisis. The performance of a thermoelectric material is quantified by the dimensionless figure of merit $ZT=\frac{S^{2}\sigma T}{\left(\kappa_{\text{e}}+\kappa_{\text{p}}\right)}$, where *S *is the Seebeck coefficient, *σ *is the electrical conductivity, *T *is the absolute temperature, and *κ*_e _and *κ*_p _are the electron- and phonon-derived thermal conductivities, respectively. An ideal thermoelectric material requires glass-like thermal transport and crystal-like electronic properties [1], i.e., one should try to improve the *ZT *value by increasing the power factor [*S*^2^*σ*] and/or decreasing the thermal conductivity (*κ *= *κ *_e _+ *κ*_p_) at an appropriate temperature. Such a task is usually very difficult since there is a strong correlation of those transport coefficients according to the Wiedemann-Franz law [2]. Low-dimensional or nanostructure approaches [3,4], however, offer new ways to effectively manipulate electron and phonon transports and thus can significantly improve the *ZT *value.

As an interesting quasi-one-dimensional nanostructure with many unusual properties, carbon nanotubes [CNTs] have attracted a lot of attention from the science community since their discovery [5]. However, few people believe that CNTs could be promising thermoelectric materials. This is probably due to the fact that although CNTs can have much higher electrical conductivities, their thermal conductivities are also found to be very high [6-11]. As a result, the *ZT *values of CNTs predicated from previous works [10,12] are rather small (approximately 0.0047). Prasheret al. [13] found the so-called 'CNT bed' structure could reduce the thermal conductivity of CNTs. However, the random network of the samples may weaken the electronic transport, and the room temperature *ZT *value is estimated to be 0.2. Jiang et al. [14] investigated the thermoelectric properties of single-walled CNTs using a nonequilibrium Green's function approach [NEGF]. They found that CNTs exhibit very favorable electronic transport properties, but the maximum *ZT *value is only 0.2 at 300 K. The possible reason is the neglect of nonlinear effect [15] in the phonon transport, and the corresponding thermal conductivity was overestimated. If the thermal conductivity can be significantly reduced without many changes to their electronic transport, CNTs may have very favorable thermoelectric properties. In this work, we use a combination of nonequilibrium molecular dynamics simulations and NEGF method to study the thermoelectric properties of a serial of CNTs with different diameters and chiralities. They are the zigzag (7,0), (8,0), (10,0), (11,0), (13,0), (14,0) and the chiral (4,2),(5,1), (6,2), (6,4), (8,4), (10,5), and all are semiconductors in their pristine form. By cooperatively manipulating the electronic and phonon transports, we shall see that these CNTs could be optimized to exhibit much higher *ZT *values by isotope substitution, isoelectronic impurities, and hydrogen adsorption. It is thus reasonable to expect that CNTs may be promising candidates for high-performance thermoelectric materials.

## Computational details

The phonon transport is studied using the nonequilibrium molecular dynamics [NEMD] simulations as implemented in the LAMMPS software package (Sandia National Laboratories, Livermore, CA, USA)[16]. The Tersoff potential [17] is adopted to solve Newtonian equations of motion according to the Müller-Plathe algorithm [18] with a fixed time step of 0.5 fs. We carry out a 300-ps constant temperature simulation and a 200-ps constant energy simulation to make sure that the system has reached a steady state. The nanotubes are then divided into 40 equal segments with periodic boundary condition, and the first and twenty-first segments are defined as the hot and cold regions, respectively. The coldest atom in the hot region and the hottest one in the cold region swap their kinetic energies every hundreds of time steps, and then temperature gradient responses and thermal flux maintain via atom interactions in neighboring segments [19,20]. The electronic transport is calculated using the NEGF method as implemented in the AtomistixToolKit code (Quantum Wise A/S, Copenhagen, Denmark) [21,22]. The nanotube is modeled by a central part connected by the left and right semi-infinite one. We use the Troullier-Martins nonlocal pseudopotentials [23] to describe the electron-ion interactions. The exchange-correlation energy is in the form of PW-91 [24], and the cutoff energy is set to be 150 Ry. We use a double ζ basis set plus polarization for the carbon atoms, and the Brillouin zone is sampled with 1 × 1 × 100 Monkhorst-Pack meshes. The mixing rate of the electronic Hamiltonian is set as 0.1, and the convergent criterion for the total energy is 4 × 10^-5^eV.

## Results and discussions

**Figure 1:**
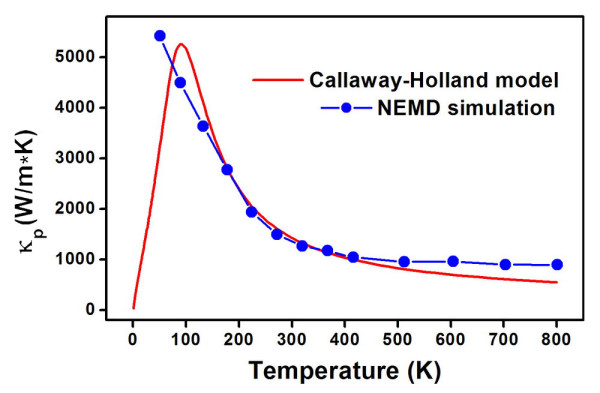
**Lattice thermal conductivity of tube (4,2) as a function of temperature**. Results from the Callaway-Holland model and the nonequilibrium molecular dynamics simulations are both shown.

We begin with the phonon transport of these CNTs using the NEMD simulations, where the phonon-induced thermal conductivity [*κ*_p_] is calculated according to Fourier's law $\kappa_{\text{p}}=\frac{J}{\left(A\cdot \nabla T\right)}$. Here, *J *is the heat flux from the hot to cold region; *A *is the cross-sectional area of the system, and ∇*T *is the temperature gradient. To test the reliability of our computational method, in Figure 1, we plot the NEMD-calculated thermal conductivity of the tube (4,2) as a function of temperature. For comparison, the result using a more accurate Callaway-Holland model [25,26] is also shown. We see that the NEMD result agrees well with that of the Callaway-Holland model when the temperature is larger than 150 K. As molecular dynamics simulation is much faster than other approaches and can handle nonlinearity when dealing with heat transport, we will use it throughout our work as long as the temperature is not very low.

For low-dimensional systems, one should pay special attention to the size effect when discussing the thermal conductivity. Both the experiment measurements [27,28] and molecular dynamics simulations [29,30] indicate that the *κ*_p _of CNTs depends on their length, which is different from that of bulk materials. Here, we use a simple approach [20] by calculating the thermal conductivity at different tube lengths and then using a linear fitting according to the formula $\frac{1}{\kappa_{\text{p}}}=a+\frac{b}{l}$. Table 1 summarizes the NEMD-calculated room temperature *κ*_p _of a series of zigzag and chiral nanotubes. It should be noted that we have carried out a quantum correction [31] to the thermal conductivity, and the tube length is assumed to be 1 μm for all the CNTs considered. As can be seen from the table, the room temperature *κ*_p _of CNTs are indeed very high which range from several hundreds to more than 1,000 W/m·K. If we focus on the zigzag CNTs, we find that the thermal conductivity decreases as the tube diameter is increased. This is also the case for the chiral CNTs with the same chiral angle (e.g., the (4,2), (8,4), and (10,5) tubes). The reason is that larger diameter CNTs have a smaller average group velocity, and the probability of the Umklapp process is higher [25,32]. On the other hand, if we focus on those CNTs with roughly similar diameters (e.g., (7,0)vs. (6,2),(11,0)vs. (8,4),(13,0)vs. (10,5)), it is interesting to find that the thermal conductivity of the chiral tube is always lower than that of the zigzag one. As these CNTs have a similar average group velocity, we believe that the more frequent phonon Umklapp scattering in the chiral tubes makes a significant contribution to the reduced thermal conductivity.

**Table 1:** Summary of the NEMD-calculated room temperature *κ*_p _of a series of zigzag and chiral nanotubes

Tubes		*d *(nm)	*θ *(°)	*κ*_p _(W/m·K)
Zigzag	(7,0)	0.548	0	1270
	(8,0)	0.627	0	955
	(10,0)	0.783	0	809
	(11,0)	0.862	0	778
	(13,0)	1.019	0	613
	(14,0)	1.097	0	599
Chiral	(4,2)	0.414	19.2	1337
	(5,1)	0.436	8.9	1413
	(6,2)	0.565	13.9	1009
	(6,4)	0.683	23.4	829
	(8,4)	0.829	19.2	721
	(10,5)	1.036	19.2	564

The corresponding tube diameter (*d*) and chiral angle (*θ*) are also given.

**Figure 2:**
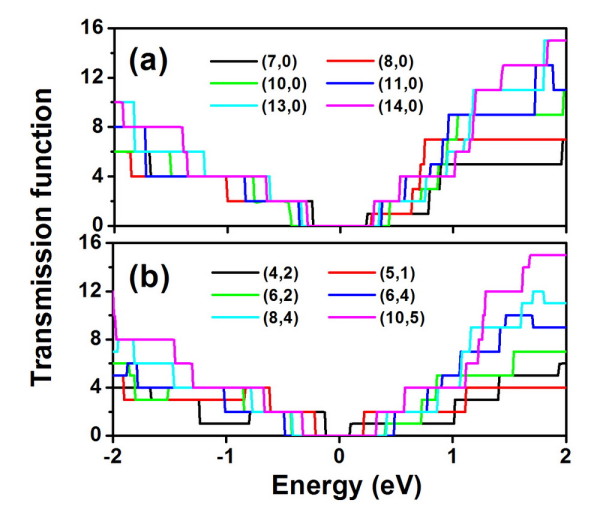
**Calculated electron transmission function for a series of (a) zigzag and (b) chiral tubes**.

**Figure 3:**
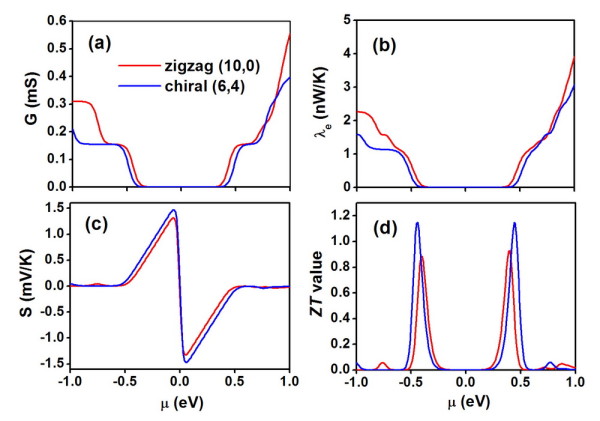
**Calculated transport coefficients at 300 K as a function of chemical potential**. These are for zigzag (10,0) and chiral (6,4). (**a**) Electric conductance, (**b**) electronic thermal conductance, and (**c**) Seebeck coefficient with (**d**) the corresponding *ZT *values.

We now move to the discussions of electronic transport using the NEGF approach. Figure 2 shows the calculated electronic transmission function [*T*(*E*)] for the above-mentioned zigzag and chiral series. Within the rigid-band picture, *E *> 0 corresponds to the *n*-type doping, while *E *< 0 corresponds to the *p*-type doping. Here, we focus on the electron ballistic transport and ignore the weak electron-phonon scattering. We see that all the investigated CNTs exhibit quantized transmission which can be essentially derived from their energy band structures. The vanishing transmission function around the Fermi level is consistent with the fact that all of them are semiconductors. It is interesting to find that those CNTs with a larger diameter have a symmetrically distributed transmission function near the Fermi level. However, this is not the case for the smaller diameter CNTs such as(7,0), (8,0), and (4,2), where we see that the number of first conduction channel is two for the *p*-type doping and one for the *n*-type doping. By integrating [33] the calculated *T*(*E*), one can easily obtain the Seebeck coefficient (*S*), the electrical conductance [*G*], and the electronic thermal conductance [*λ*_e_] within the linear response limit. Here, we choose the zigzag (10,0) and chiral (6,4) as two typical examples and plot in Figure 3 the corresponding transport coefficients at 300 K as a function of chemical potential [*μ*]. Note that the chemical potential indicates the doping level or carrier concentration of the system, and the *n*-type doping corresponds to *μ *> 0, while *p*-type corresponds to *μ *< 0. As can be seen in Figure 3a, b, both *G *and *λ*_e _of these two CNTs vanish around the Fermi level (*μ *= 0) since this area corresponds to the band gap of the systems. When the chemical potential moves to the edge of the first conduction channels, there is a sharp increase of *G *and *λ*_e _For both the (10,0) and (6,4) tubes, the *S *shown in Figure 3c is rather symmetric about the Fermi level, which can be attributed to the symmetrically distributed first conduction channels (see Figure 2). The absolute value of the Seebeck coefficient reaches the maximum value at μ ≈ ± *κ*_B_*T *and then decreases until vanish near the edge of band gap.

It should be mentioned that we have used the term 'conductivity' for the phonon transport but 'conductance' for the electronic transport. To avoid arbitrary definition of cross-sectional area in low-dimensional system such as CNTs, we rewrite the figure of merit as $ZT=\frac{S^{2}GT}{\left(\lambda_{\text{e}}+\lambda_{\text{p}}\right)}$, where the phonon-induced thermal conductance (*λ*_p_) has been used to replace the original thermal conductivity(*κ*_p_). Figure 3d shows the chemical potential dependent *ZT *value at 300 K for the (10,0) and (6,4) tubes. We see that both of them exhibit two peak values around the Fermi level, which suggest that by appropriate *p*-type and *n*-type doping, one can significantly enhance the thermoelectric performance of CNTs. For the (10,0) tube, the maximum *ZT *value is found to be 0.9, and it appears at *μ *= ± 0.40 eV. In the case of (6,4) tube, the *ZT *value can be optimized to 1.1 at μ = ± 0.44 eV. The same doping level for the *p*-type and *n*-type doping in the (10,0) or (6,4) tubes is very beneficial for their applications in real thermoelectric devices.

**Figure 4:**
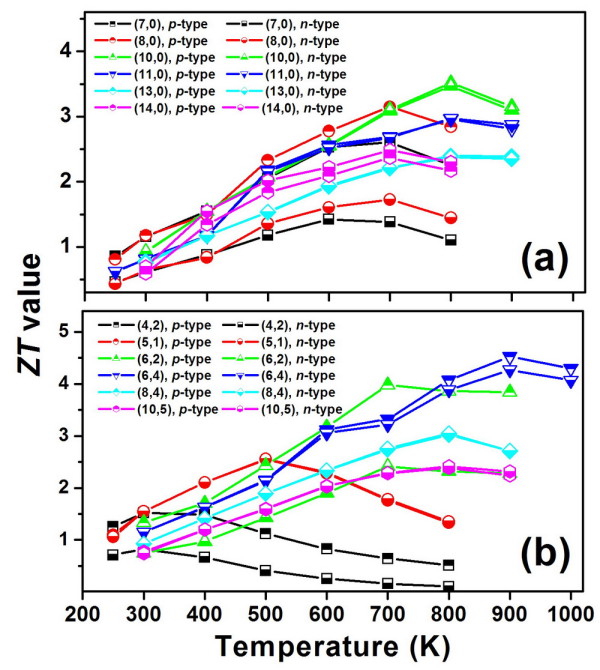
**Optimized *ZT *values as a function of temperature**. These are for a series of (**a**) zigzag and (**b**) chiral tubes. The results for the *p*-type and *n*-type doping are both shown.

Up to now, we are dealing with room temperature, and the corresponding *ZT *values are still not comparable to that of the best commercial materials. Moreover, a thermoelectric material may be needed to operate at different temperatures for different applications. We thus perform additional transport calculations where the temperature ranges from 250 to 1,000 K. Figure 4 plots the calculated *ZT *values as a function of temperature for the above-mentioned zigzag and chiral series. At each temperature, two *ZT *values are shown which correspond to the optimized *p*-type and *n*-type doping in each tube. Except for the small (4,2) tube with a maximum *ZT *value at 300 K, we see in Figure 4 that the thermoelectric performance of other CNTs can be significantly enhanced at a relatively higher temperature. The maximum *ZT *values achieved are 3.5 for the zigzag (10,0) at 800 K and 4.5 for the chiral (6,4) at 900 K. These values are very competitive with that of conventional refrigerators or generators. It is interesting to note that among the investigated CNTs, both the (10,0) and (6,4) tubes have an intermediate diameter (0.7 to 0.8 nm), and those with larger or smaller diameters have a relatively less favorable thermoelectric performance. On the other hand, we see that almost all the zigzag tubes exhibit a peak *ZT *value at an intermediate temperature (700 to 800 K). In contrast, the peak for the chiral series moves roughly from 300 to 900 K as the tube diameter is increased. Our calculated results thus provide a simple map by which one can efficiently find the best CNT for the thermoelectric applications at different operating temperatures.

**Figure 5:**
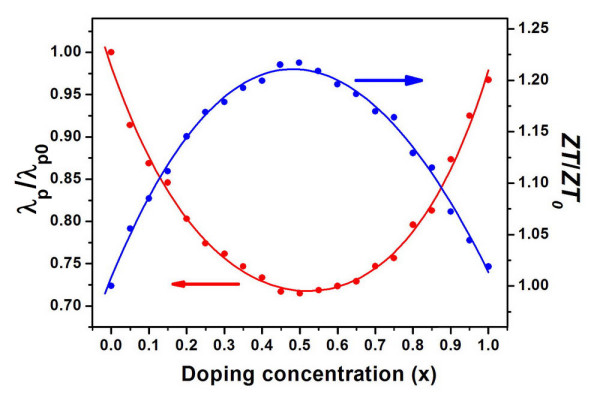
**Lattice thermal conductance and the *ZT *value**. Calculated lattice thermal conductance (red) and optimized *ZT *value (blue) at 800 K for the (10,0) tube, where the ^12^C atoms are substituted by ^13^C atoms with different concentrations. Note that the results are given with respect to those of the pure ^12^C tube.

To further improve the thermoelectric performance of these CNTs, we have considered isotope substitution which is believed to reduce the phonon-induced thermal conductance without changing the electronic transport properties [34-36]. Here, we choose (10,0) as an example since it has the highest *ZT *value among those in the zigzag series, and the zigzag tubes are usually easier to be fabricated in or to be selected from the experiments than the chiral ones. In our calculations, the ^12^C atoms in the (10,0) tube are randomly substituted by^13^C atoms at different concentrations. The corresponding lattice thermal conductance as well as the *ZT *value at 800 K is shown in Figure 5 with respect to the pristine values. Due to the mass difference between ^12^C and ^13^C, we see that the calculated thermal conductance of the (10,0) tube decreases with the increasing concentration of ^13^Catoms. Of course, if half or more ^12^Catoms are substituted, the situation is reversed. The thermal conductance can be well fitted by a double exponential function $\frac{\lambda_{\text{p}}}{\lambda_{\text{p0}}}=0.36e^{\frac{-x}{0.28}}+0.35e^{\frac{-\left(1-x\right)}{0.24}}+0.62$, where *x *is the concentration of ^13^C atoms. For a light isotope substitution (^12^C_0.95_^13^C_0.05_), the thermal conductance is already reduced by about 9% and the *ZT *value can be increased to 3.7 from the pristine value of 3.5. If half ^12^C atoms are replaced (^12^C_0.5_^13^C_0.5_), the corresponding thermal conductance reaches the minimum and the *ZT *value can be as high as 4.2, which suggests its appealing thermoelectric applications.

**Figure 6:**
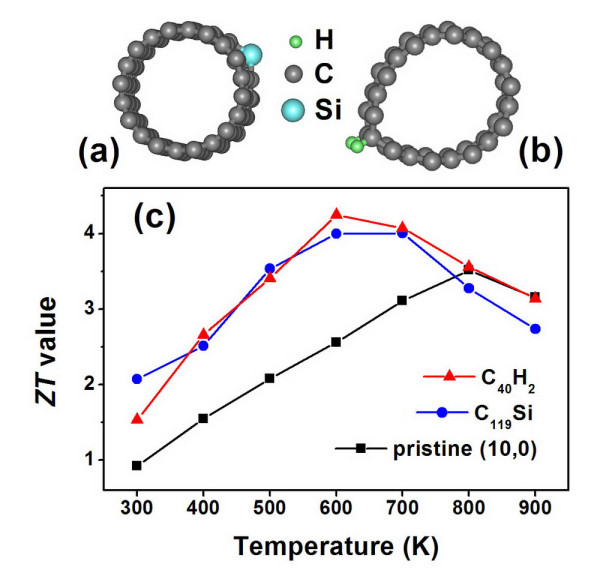
**Fully relaxed structures and optimized *ZT *values of CNTs**. Top view of the fully relaxed structures for (**a**) Si-doped (10,0) tube with nominal formula of C_119_Si and (**b**) the (10,0) tube chemisorbed with hydrogen atoms having nominal formula of C_40_H_2_. (**c**) Plots of the optimized *ZT *value as a function of temperature for theC_119_Si and C_40_H_2 _products, and the results for the pristine (10,0) tube is also shown for comparison.

Introducing isoelectronic impurities is another effective way to localize phonon and reduce lattice thermal conductance due to impurity scattering [37]. Here, we choose Si as an example and consider a very low concentration where one C atom in a (10,0) supercell containing three primitive cells is replaced by a Si atom. The resulting product has a nominal formula of C_119_Si and is schematically shown in Figure 6a. As the mass difference between C and Si is even larger, we find that the phonon-derived thermal conductance of C_119_Si is significantly reduced by 45% to 60% compared with that of the pristine(10,0) tube in the temperature range from 300 to 900 K. On the other hand, since C and Si atoms have the same electron configuration, one may expect that Si doping will not change much of the electronic transport properties. Indeed, our calculations only find a small weakening of the power factor (*S*^2^*G*). As a result, we see in Figure 6c that there is an overall increase of the *ZT *value at the temperature range of 300 to 700 K. The Si-doped product has a maximum *ZT *= 4.0 at *T *= 600 K compared with the pristine value of 3.5 at *T *= 800 K. It is worth to mention that in a wide temperature range (450 to 800 K), the *ZT *values of the Si-doped product are all higher than 3.0 which is very beneficial for their thermoelectric applications.

A similar improvement of the thermoelectric performance can be achieved by hydrogen adsorption on the (10,0) tube. As shown in Figure 6b, two hydrogen atoms are chemisorbed on top of a C-C bond along the tube axis, and the product has a concentration of C_40_H_2_. Our calculated results indicate that such hydrogen adsorption causes deformation of the (10,0) tube and reduces both the phonon- and electron-induced thermal conductance while keeping the *S*^2^*G *less affected. For example, the calculated *λ*_p _at 600 K is 0.072 nW/K, which is much lower than that found for the pristine (10,0) tube (0.21 nW/K). The calculated *λ*_e _also decreases from 0.089 to 0.062 nW/K. At the same time, we find that the *S*^2^*G *of the chemisorbed product (9.47 × 10^-13 ^W/K^2^) is slightly lower than that of the pristine (10,0) tube (1.28 × 10^-12 ^W/K^2^). As a result, the calculated *ZT *value at 600 K increases significantly from 2.6 to 4.2 which is even higher than the highest value of the pristine (10,0) tube. The chemisorptions of hydrogen also increase the *ZT *value at other temperatures, as indicated in Figure 6c. It is interesting to note that the temperature-dependent behavior almost coincides with that from Si doping, especially in the temperature region from 400 to 700 K.

## Summary

In summary, our theoretical calculations indicate that by appropriate *n*-type and *p*-type doping, one can obtain much higher *ZT *values for both the zigzag and armchair CNTs, and those tubes with an intermediate diameter (0.7 to 0.8 nm) seems to have better thermoelectric properties than others. With the zigzag (10,0) as an example, we show that the phonon-induced thermal conductance can be effectively reduced by isotope substitution, isoelectronic impurities, and hydrogen adsorption, while the electronic transport is less affected. As a result, the *ZT *value can be further enhanced and is very competitive with that of the best commercial materials. To experimentally realize this goal, one needs to fabricate CNTs with specific diameter and chirality, and the tube length should be at least 1 μm. This may be challenging but very possible, considering the fact that the (10,0) tube was successfully produced by many means, such as by direct laser vaporization [38], electric arc technique [39], and chemical vapor deposition [40], and can be selected from mixed or disordered samples using a DNA-based separation process [41].

## Competing interests

The authors declare that they have no competing interests.

## Authors' contributions

XJT carried out the NEGF and NEMD calculations. HJL participated in the design of the study and discussions of the theoretical results. YWW, HYL, and LP participated in the implementation of the LAMMPS and ATK codes. JS and XFT participated in the discussions of related experimental works. All authors read and approved the final manuscript.
